# Supplementary material for: Use of Enzymatically Activated Carbon Monoxide Donors for Sensitizing Drug-Resistant Tumor Cells
Source: Int J Mol Sci. 2023 Jul 9;24(14):11258. doi: 10.3390/ijms241411258 (PMC10379931; doi:10.3390/ijms241411258)

Supporting Information

Use of Enzymatically Activated Carbon Monoxide Donors for Sensitizing Drug-Resistant Tumor Cells

Federica Sodano [1], Barbara Rolando [2], Loretta Lazzarato [2], Costanzo Costamagna [3], Mariacristina Failla [2], Chiara Riganti [3] and Konstantin Chegaev [2,*]

[1] Department of Pharmacy, “Federico II” University of Naples, 80131 Naples, Italy; federica.sodano@unina.it
[2] Department of Drug Science and Technology, University of Torino, 10125 Torino, Italy; barbara.rolando@unito.it (B.R.); loretta.lazzarato@unito.it (L.L.); mariacristina.failla@unito.it (M.F.)
[3] Department of Oncology, University of Torino, 10125 Torino, Italy; costanzo.costamagna@unito.it (C.C.); chiara.righanti@unito.it (C.R.)

Corresponding author: konstantin.chegaev@unito.it; Tel.: +39-0116707140

Table of contents

Figure S1. Degradation of compound 1 in human serum ……………………………………………….S2

Figure S2. Mitochondrial parameters and caspases activation in triple negative breast cancer cells treated with compound 1 and docetaxel…………………………………………………………..S3

Figure S3. Mitochondrial and total ROS and lipoperoxidation in triple negative cancer cell lines treated with compound 1 and docetaxel …………………………………………………………….S4

NMR characterization of compounds **1**, **4** – **12** ………………………………………………………S5-S11

MS spectra of compounds **1**, **4** – **12** ……………………………………………………………….S12-S15

A

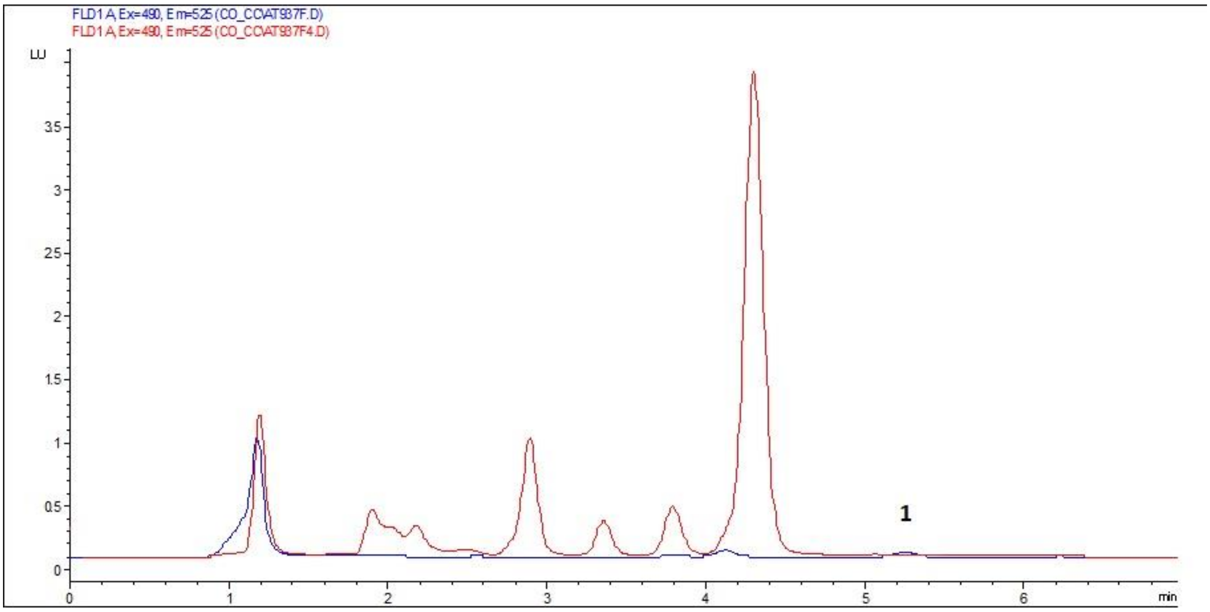

B

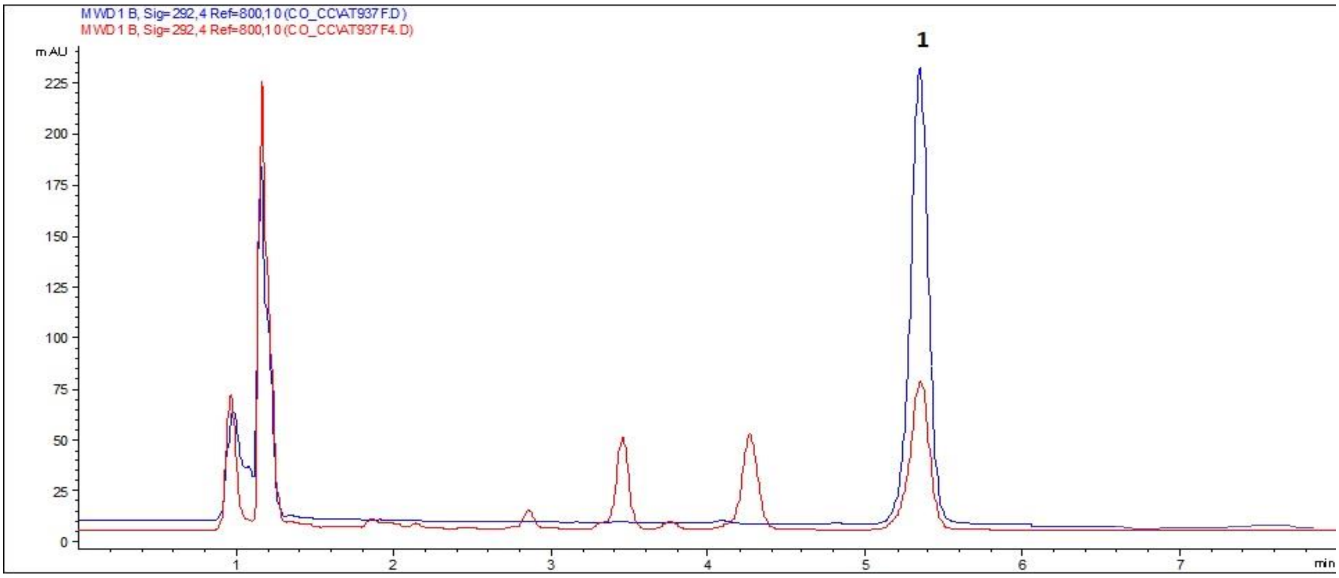

Figure S1. Degradation of compound **1** in human serum: chromatogram of **1** at time 0 (blue track) and after 180 min (red track) of incubation at 37°C in human serum;
(**A**) Fluorescence detection (exc. 490, emiss. 525 nm); (**B**) UV detection at 292 nm (ref 800 nm).

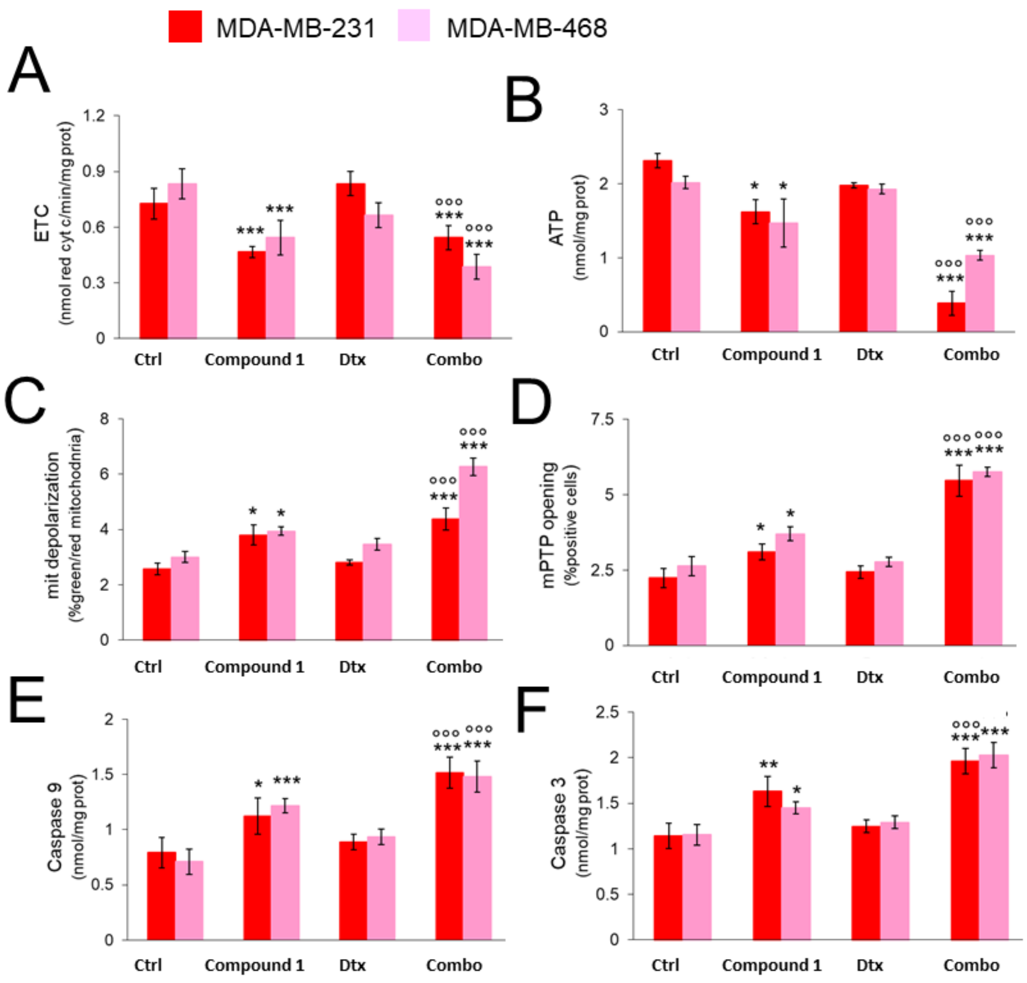

Figure S2. Mitochondrial parameters and caspases activation in triple negative breast cancer cells treated with compound 1 and docetaxel. Human triple negative breast cancer MDA-MB-231 and MDA-MB-468 cells were treated 24 h with 1 µM compound 1+1 µM docetaxel (Dtx), alone or in association (Combo). Ctrl: cells grown in fresh medium. (**A**) Electron transport chain measured spectrophotometrically. (**B**) Mitochondria ATP measured by chemiluminescence-based assay. (**C-D**) Percentage of mitochondria with depolarized membrane, as index of mitochondrial damage, assessed fluorometrically (**C**), and characterized by open mitochondrial permeability transition pore (mPTP), measured by flow cytometry (**D**). (**E-F**) Activity of caspase-9 (**E**) and caspase-3 (**F**), measured fluorometrically. All assays were performed in triplicate (n=3 biological replicates). Data are means ± SD. **p*<0.05, **p<0.01, ****p*<0.001: compound 1+chemotherapeutic drug versus untreated cells; °°°*p*<0.001: compound 1+chemotherapeutic drug versus chemotherapeutic drug alone.

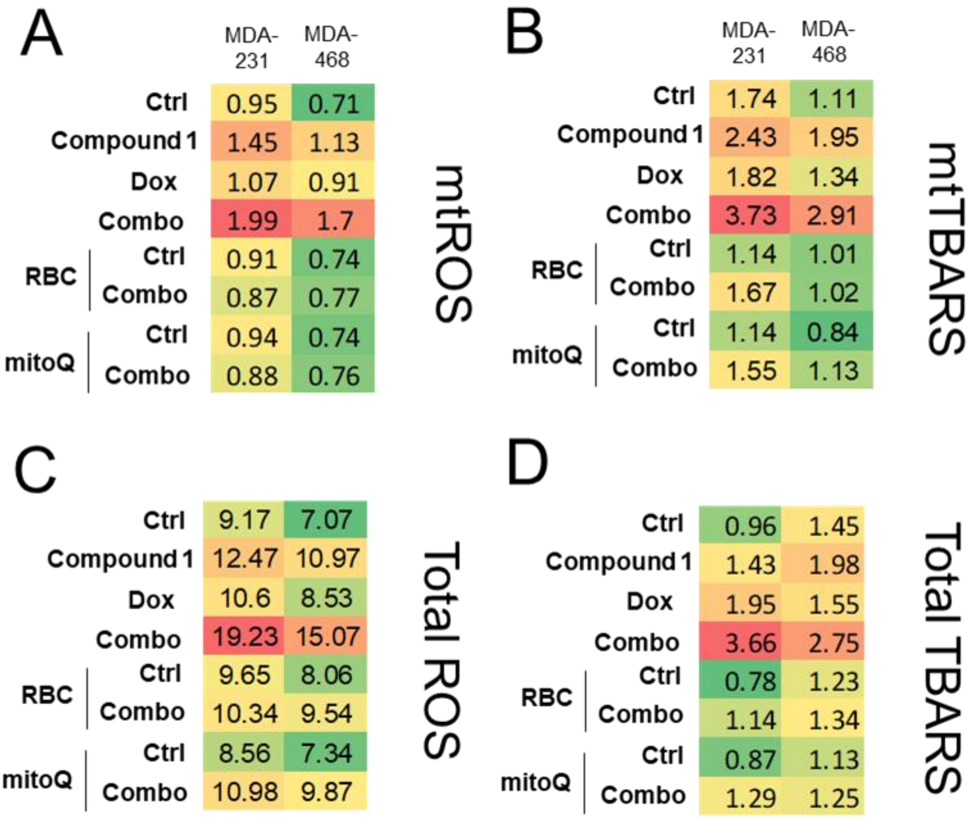

Figure S3. Mitochondrial and total ROS and lipoperoxidation in triple negative cancer cell lines treated with compound 1 and docetaxel. Human triple negative breast cancer MDA-MB-231 and MDA-MB-468 cells were treated 24 h with 1 µM compound 1+1 µM docetaxel (Dtx), alone or in association (Combo). Ctrl: cells grown in fresh medium. When indicated, 10 µg/mL packed red blood cells (RBC), as a CO scavenger, or 0.4 µM mitoquinol (mitoQ), as a mitochodrial ROS scavenger, were co-incubated. (**A-B**) ROS, as an index of oxidative stress, measured fluorometrically and TBARS, as an index of lipoperoxidation, determined spectrophotometrically in extracted mitochondria. (**C-D**) ROS and TBARS measured in whole cell lysate. All assays were performed in triplicate (n=3 biological replicates). Data are represented as heatmap (numbers correspond to means). For all panels: $^{**}p<0.01$: compound 1 versus untreated cells, $^{***}p<0.001$: compound 1+chemotherapeutic drug versus untreated cells; $^{\circ\circ\circ}p<0.001$: compound 1+chemotherapeutic drug versus chemotherapeutic drug alone; $^{\S\S\S}p<0.001$: compound 1+chemotherapeutic drug versus compound 1 alone; $^{\#\#\#}p<0.001$: RBC+Combo/mitoQ+Combo versus Combo alone.

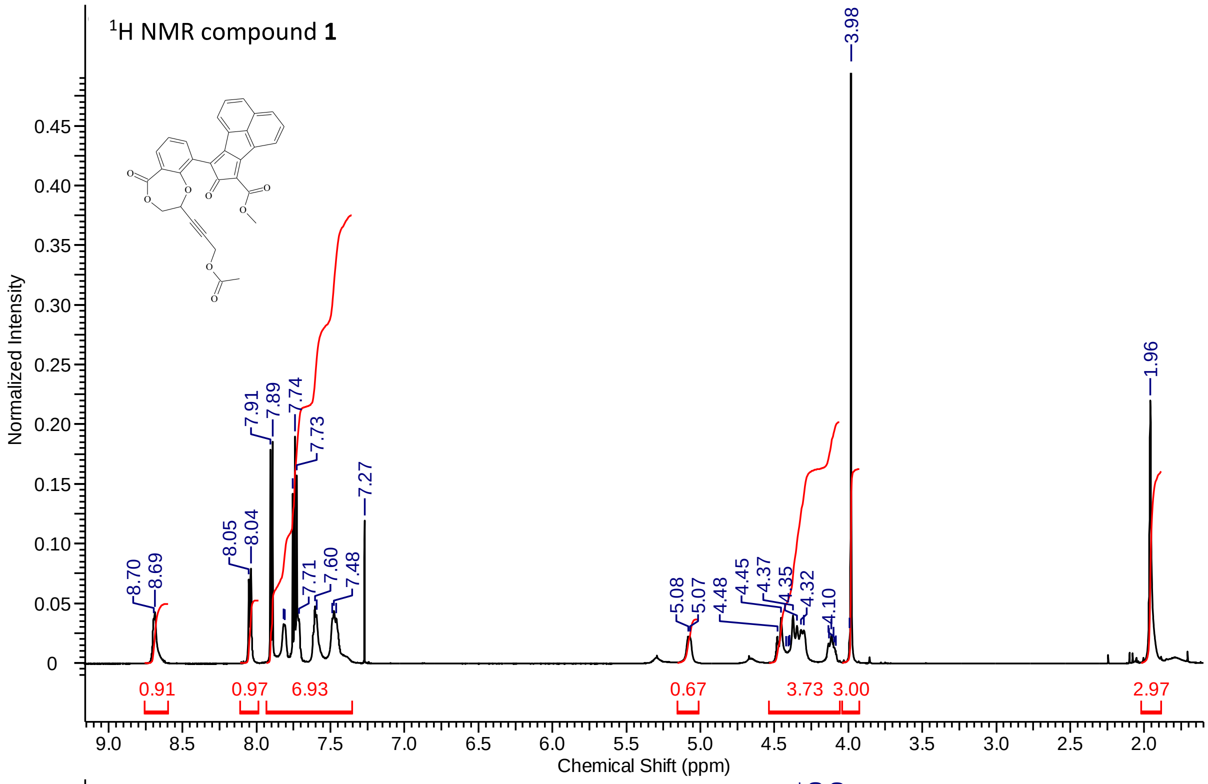

^{1}H NMR compound **1**
Normalized Intensity
Chemical Shift (ppm)
8.70 8.69 8.05 8.04 7.91 7.89 7.74 7.73 7.71 7.60 7.48 7.27 5.08 5.07 4.48 4.45 4.37 4.35 4.32 4.10 3.98 1.96
0.91 0.97 6.93 0.67 3.73 3.00 2.97

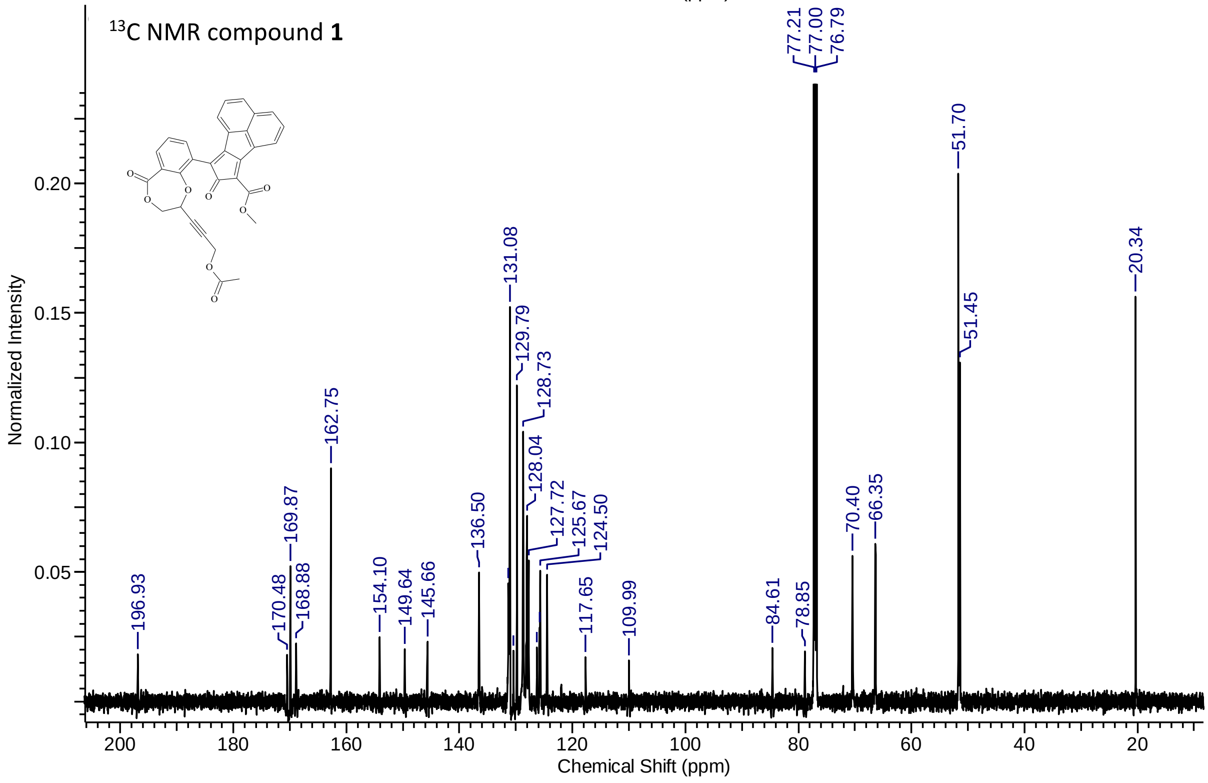

^{13}C NMR compound **1**
Normalized Intensity
Chemical Shift (ppm)
196.93 170.48 169.87 168.88 162.75 154.10 149.64 145.66 136.50 131.08 129.79 128.73 128.04 127.72 125.67 124.50 117.65 109.99 84.61 78.85 77.21 77.00 76.79 70.40 66.35 51.70 51.45 20.34

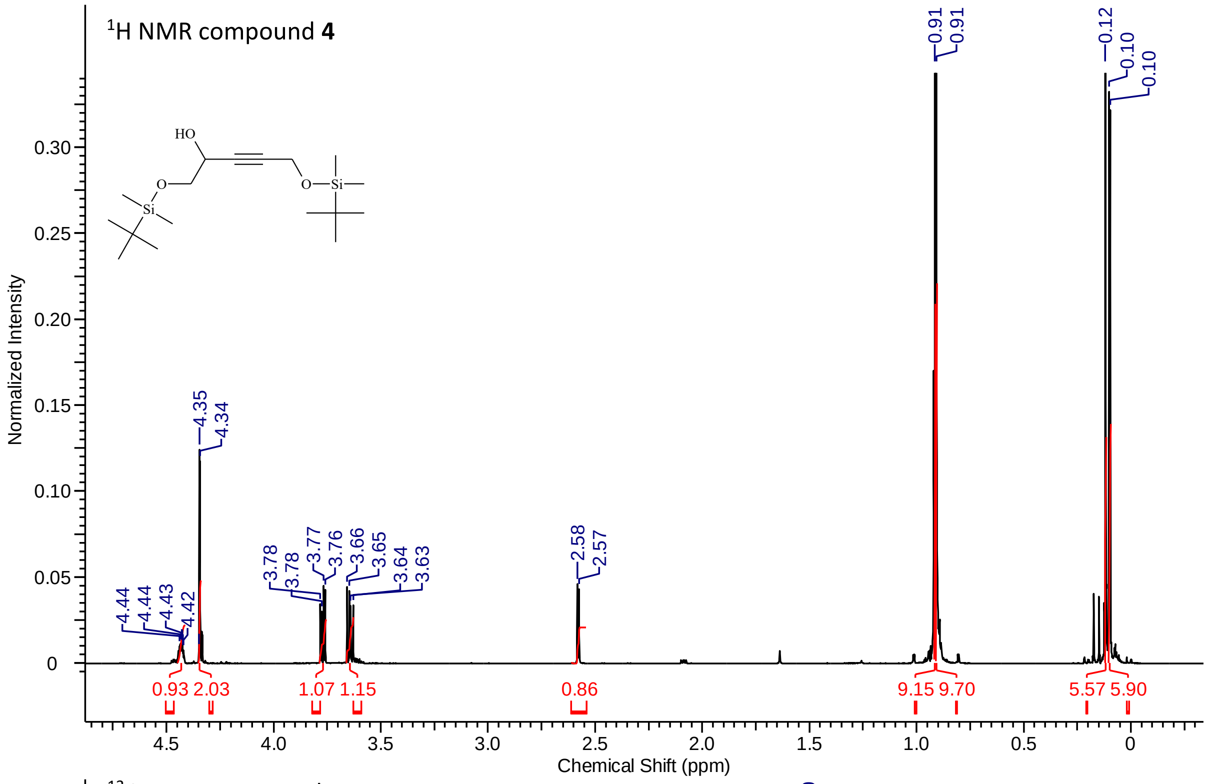

^{1}H NMR compound **4**
Normalized Intensity
Chemical Shift (ppm)

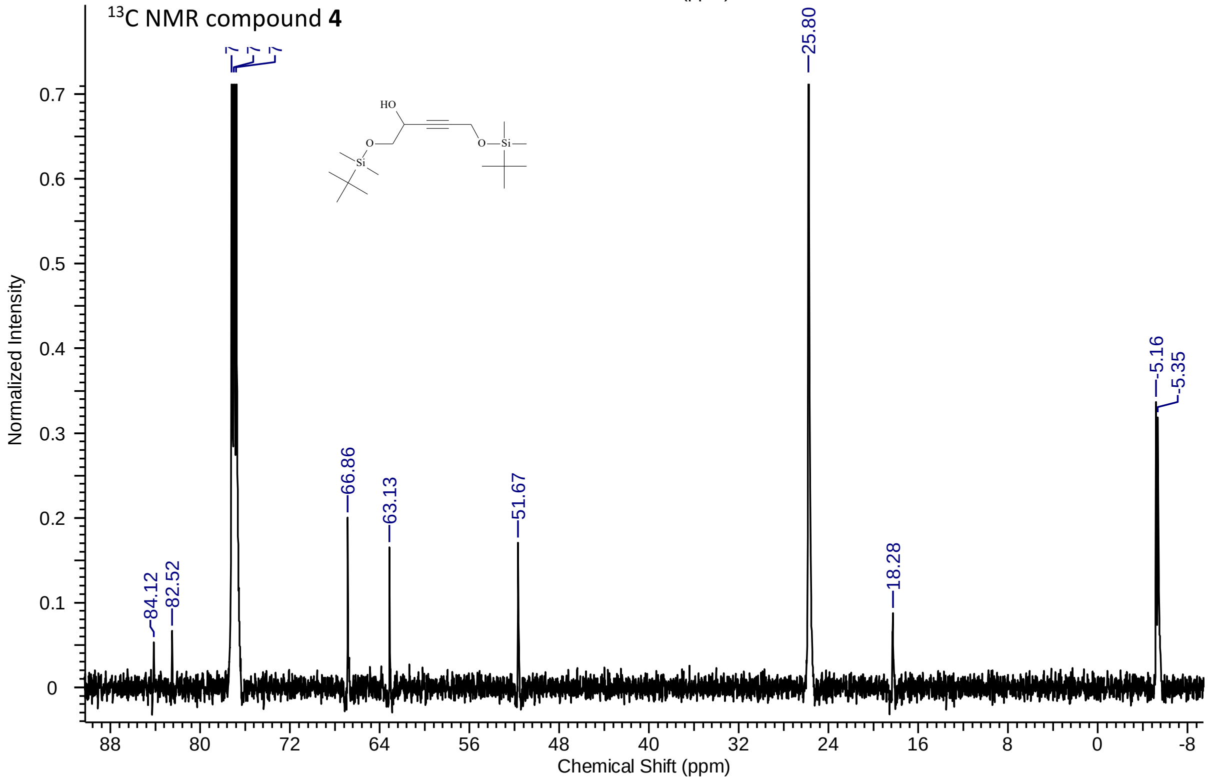

^{13}C NMR compound **4**
Normalized Intensity
Chemical Shift (ppm)

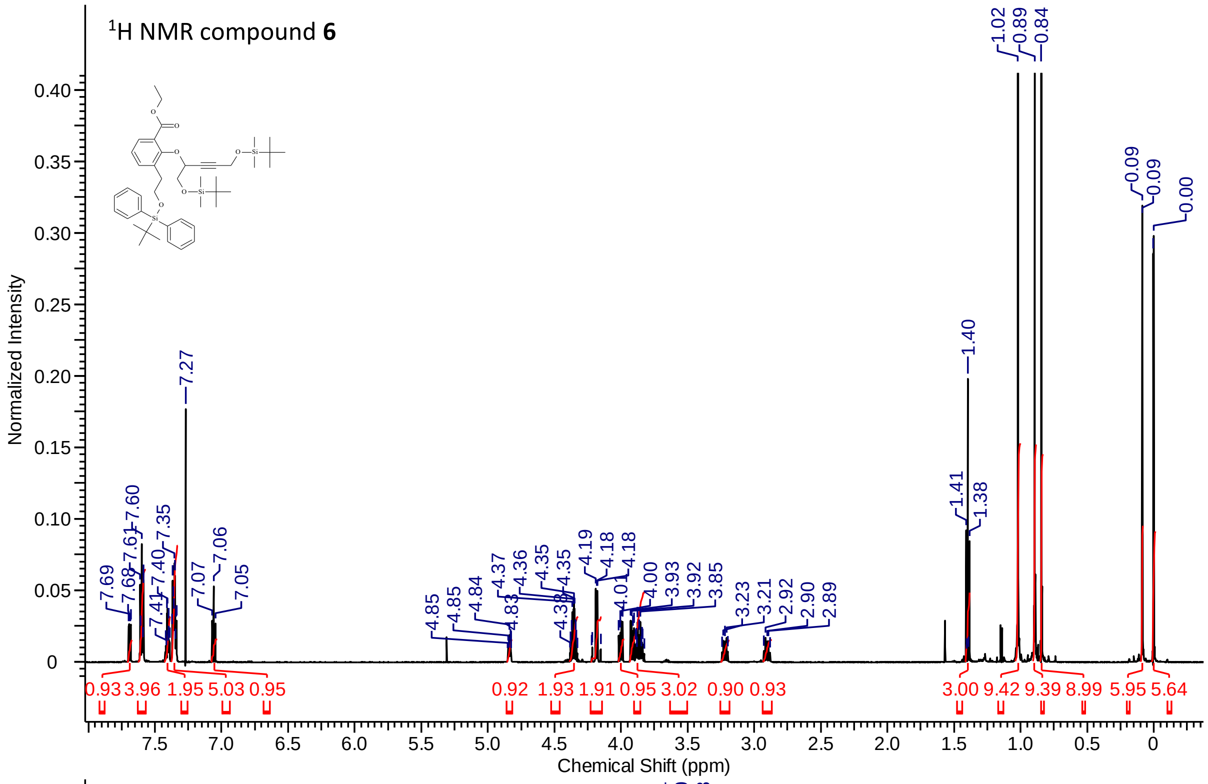

^{1}H NMR compound **6**
Normalized Intensity
Chemical Shift (ppm)

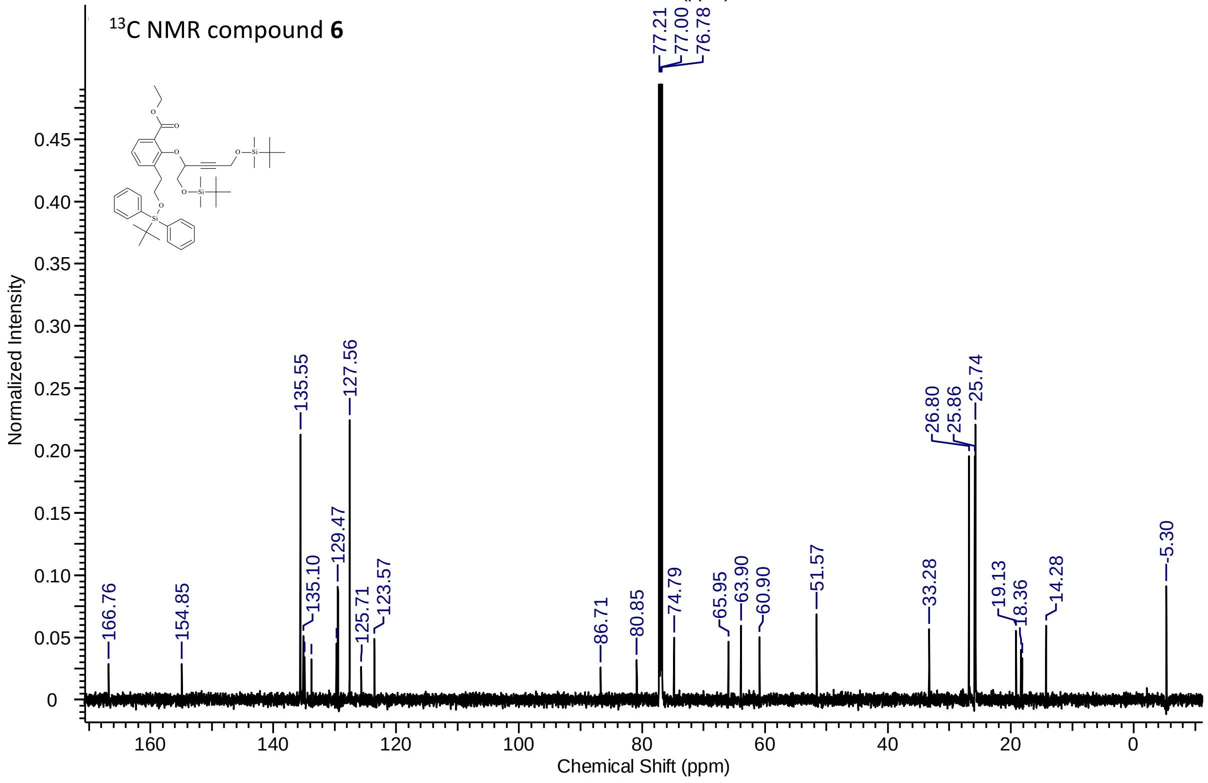

^{13}C NMR compound **6**
Normalized Intensity
Chemical Shift (ppm)

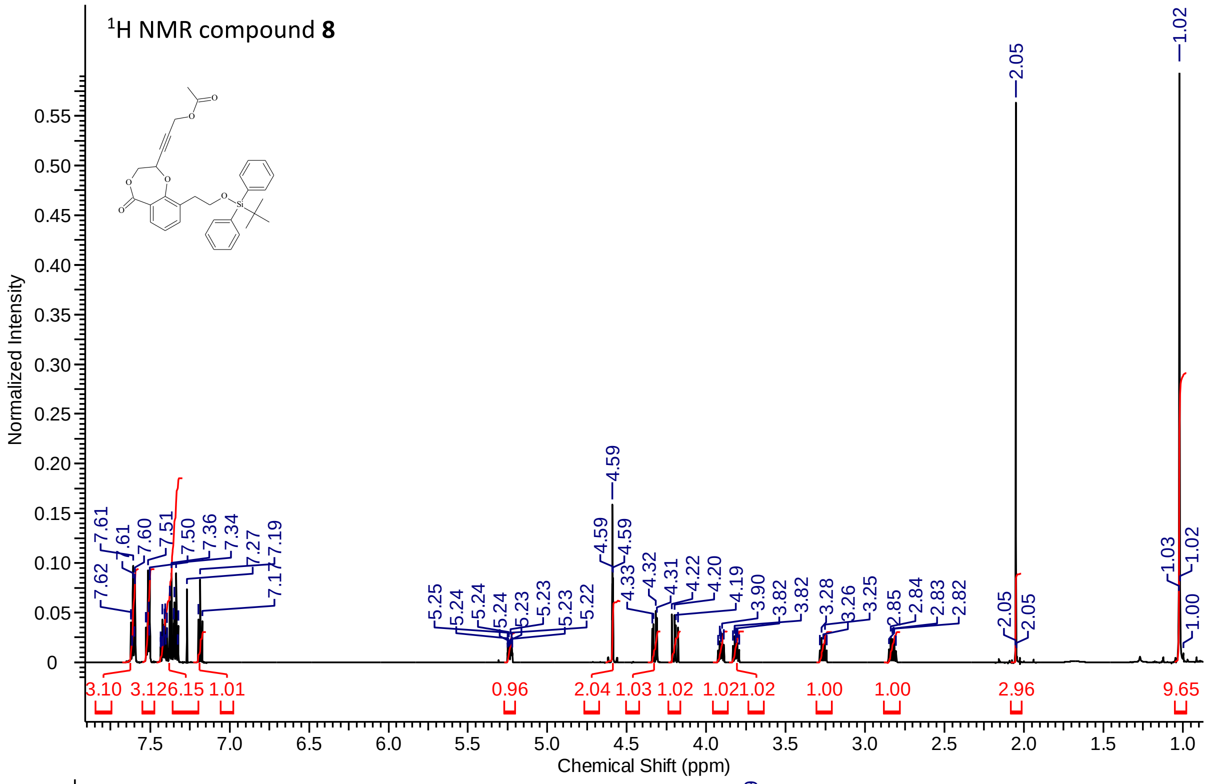

^{1}H NMR compound **8**
Normalized Intensity
Chemical Shift (ppm)
7.62 7.61 7.61 7.60 7.51 7.50 7.36 7.34 7.27 7.19 7.17
5.25 5.24 5.24 5.24 5.23 5.23 5.23 5.22
4.59 4.59 4.59 4.33 4.32 4.31 4.22 4.20 4.19 3.90 3.82 3.82 3.28 3.26 3.25 2.85 2.84 2.83 2.82 2.05 2.05 2.05 1.03 1.02 1.02 1.00
3.10 3.12 6.15 1.01 0.96 2.04 1.03 1.02 1.02 1.02 1.00 1.00 2.96 9.65

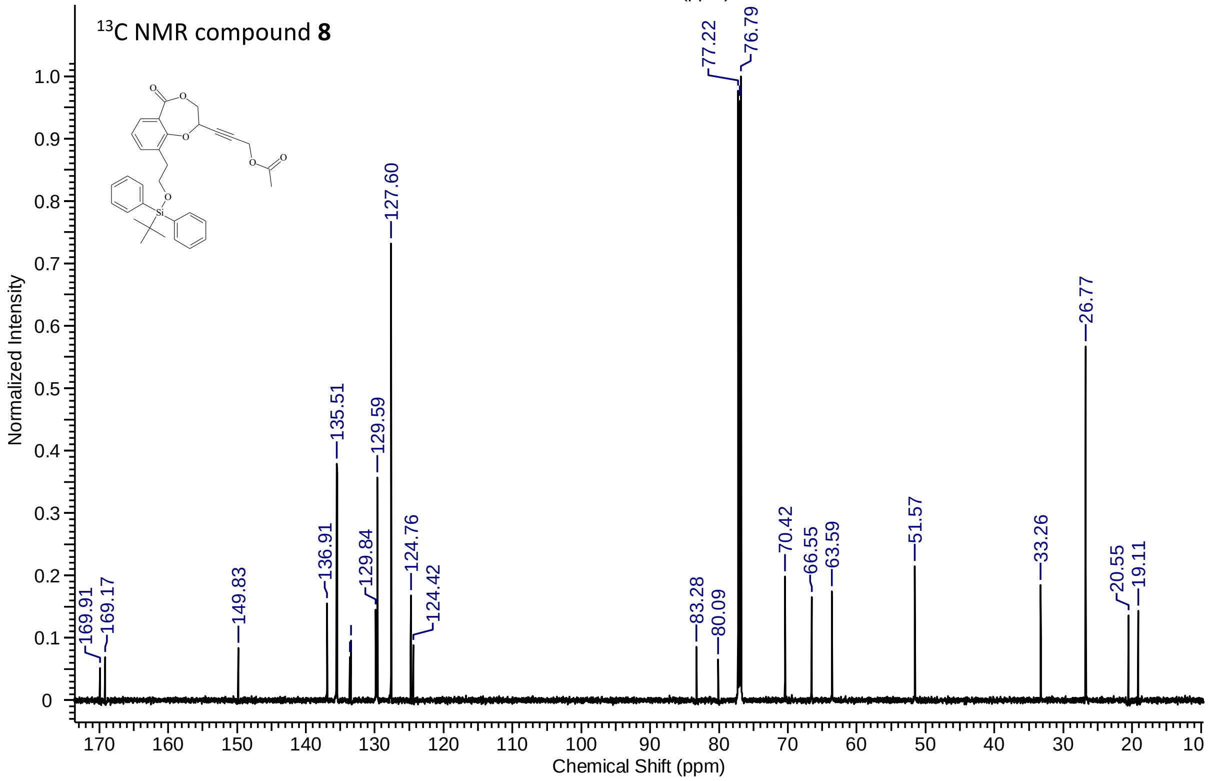

^{13}C NMR compound **8**
Normalized Intensity
Chemical Shift (ppm)
169.91 169.17 149.83 136.91 135.51 129.84 129.59 127.60 124.76 124.42 83.28 80.09 77.22 76.79 70.42 66.55 63.59 51.57 33.26 26.77 20.55 19.11

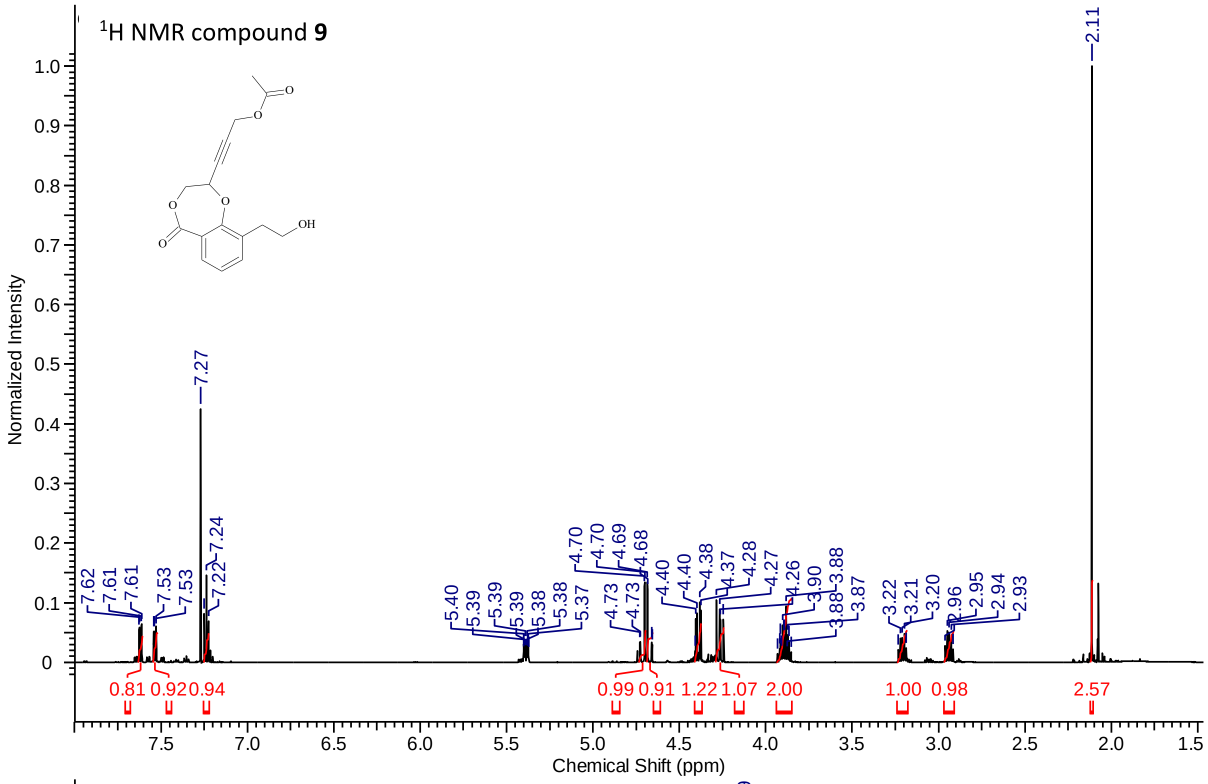

^{1}H NMR compound **9**
OH
Normalized Intensity
Chemical Shift (ppm)

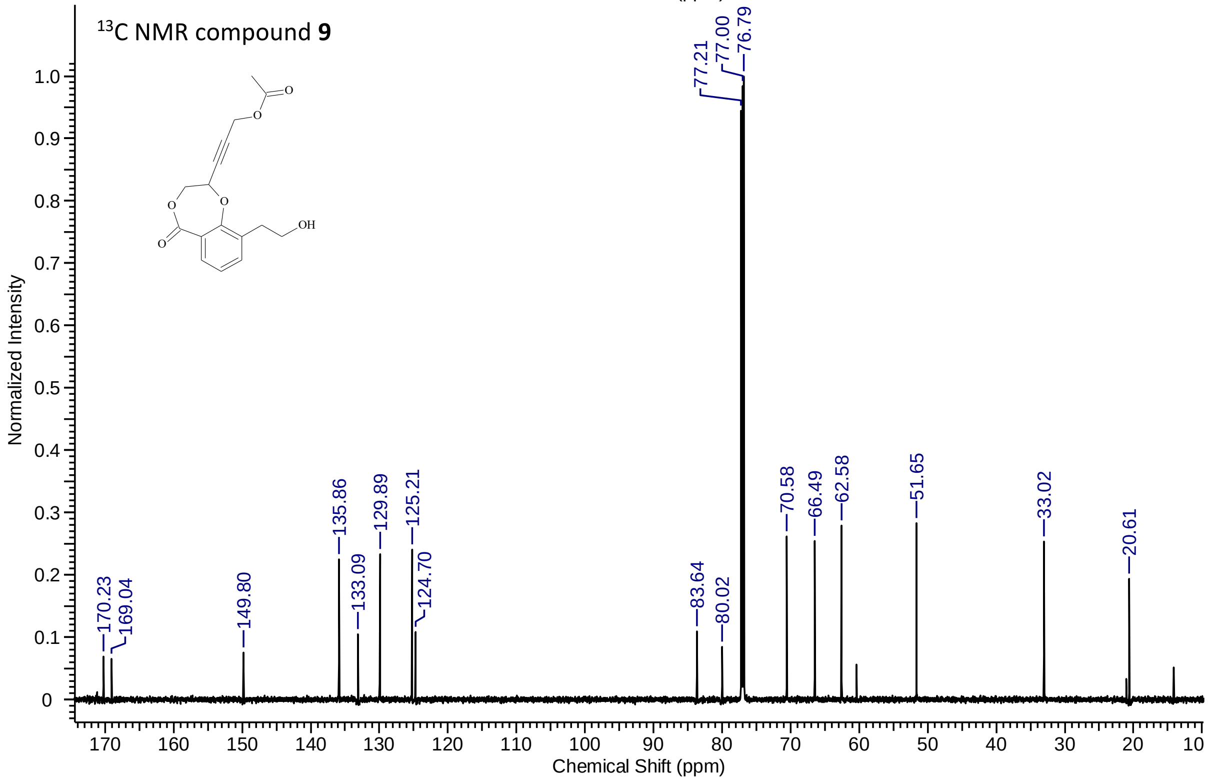

^{13}C NMR compound **9**
OH
Normalized Intensity
Chemical Shift (ppm)

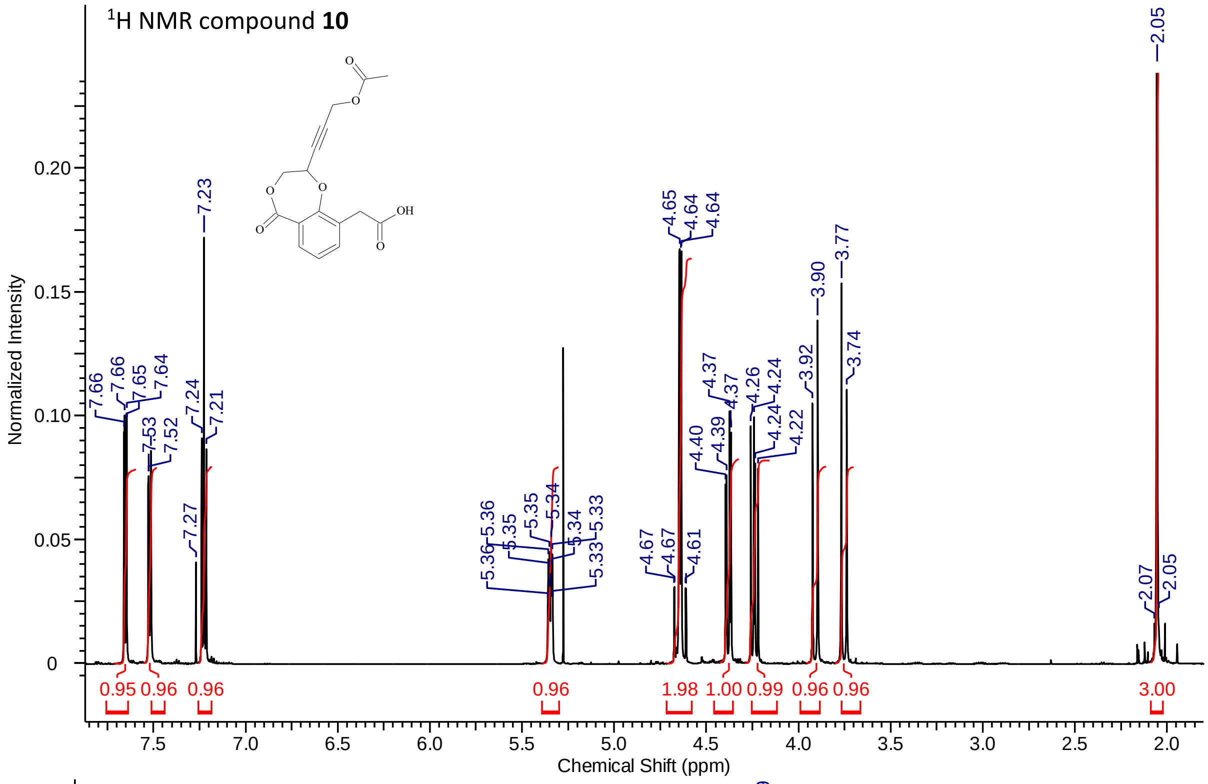

^1H NMR compound **10**
Normalized Intensity
Chemical Shift (ppm)
7.66
7.66
7.65
7.64
7.53
7.52
7.27
7.24
7.23
7.21
5.36
5.36
5.35
5.35
5.34
5.34
5.33
5.33
4.67
4.67
4.65
4.64
4.64
4.61
4.40
4.39
4.37
4.37
4.26
4.24
4.24
4.22
3.92
3.90
3.77
3.74
2.07
2.05
2.05
0.95
0.96
0.96
0.96
1.98
1.00
0.99
0.96
0.96
3.00
OH

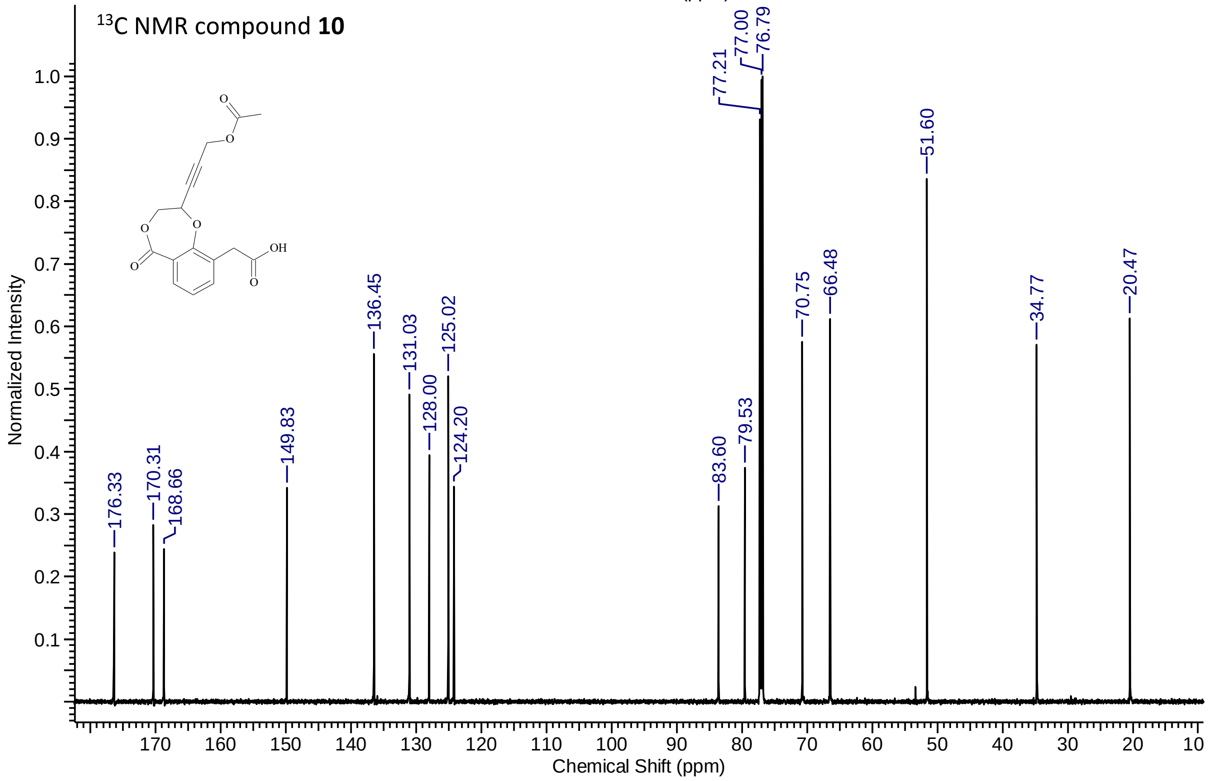

^{13}C NMR compound **10**
Normalized Intensity
Chemical Shift (ppm)
176.33
170.31
168.66
149.83
136.45
131.03
128.00
125.02
124.20
83.60
79.53
77.21
77.00
76.79
70.75
66.48
51.60
34.77
20.47
OH

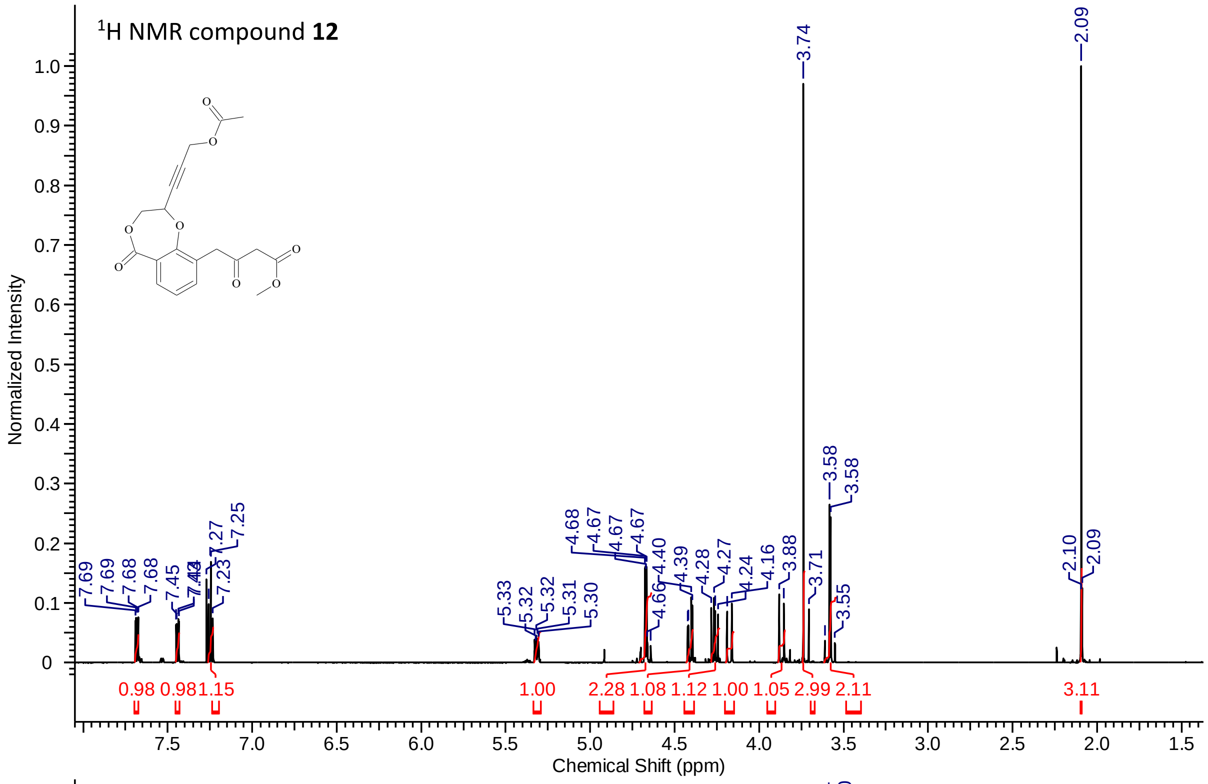

^{1}H NMR compound **12**
Normalized Intensity
Chemical Shift (ppm)

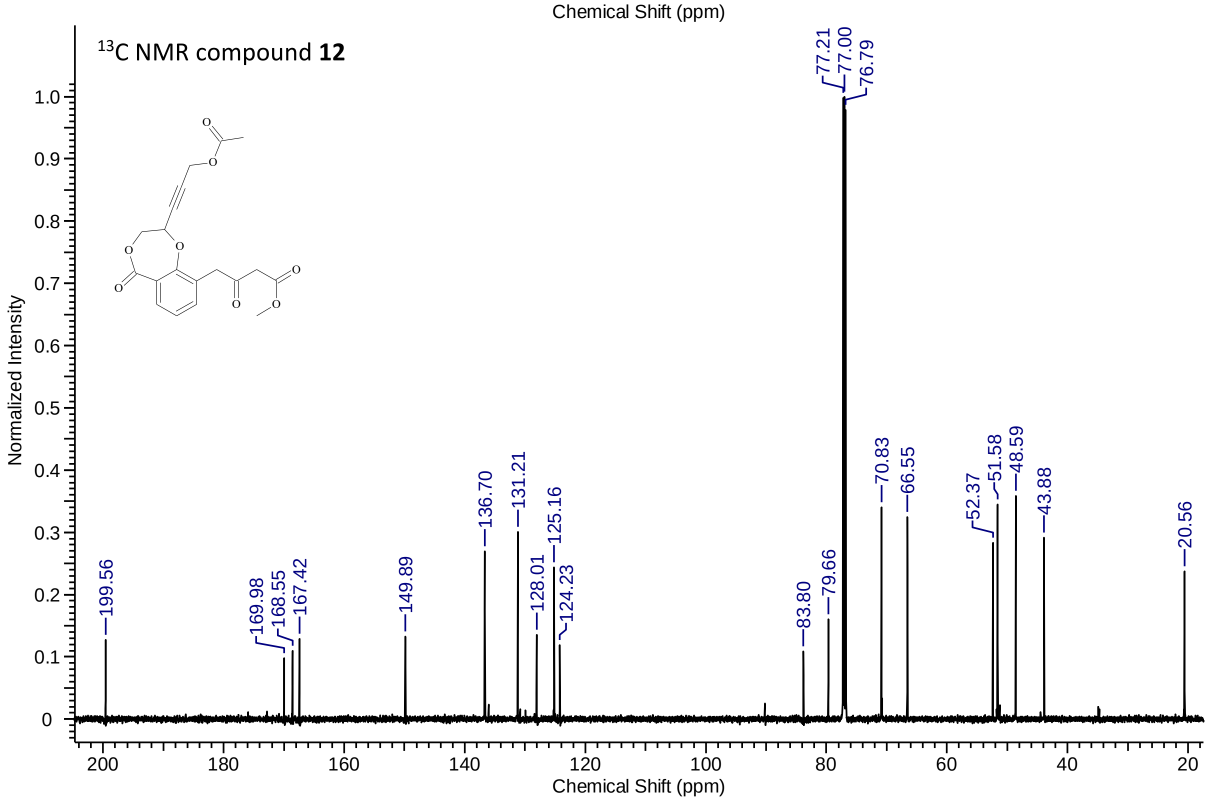

^{13}C NMR compound **12**
Normalized Intensity
Chemical Shift (ppm)

MS ESI$^+$ compound **1**

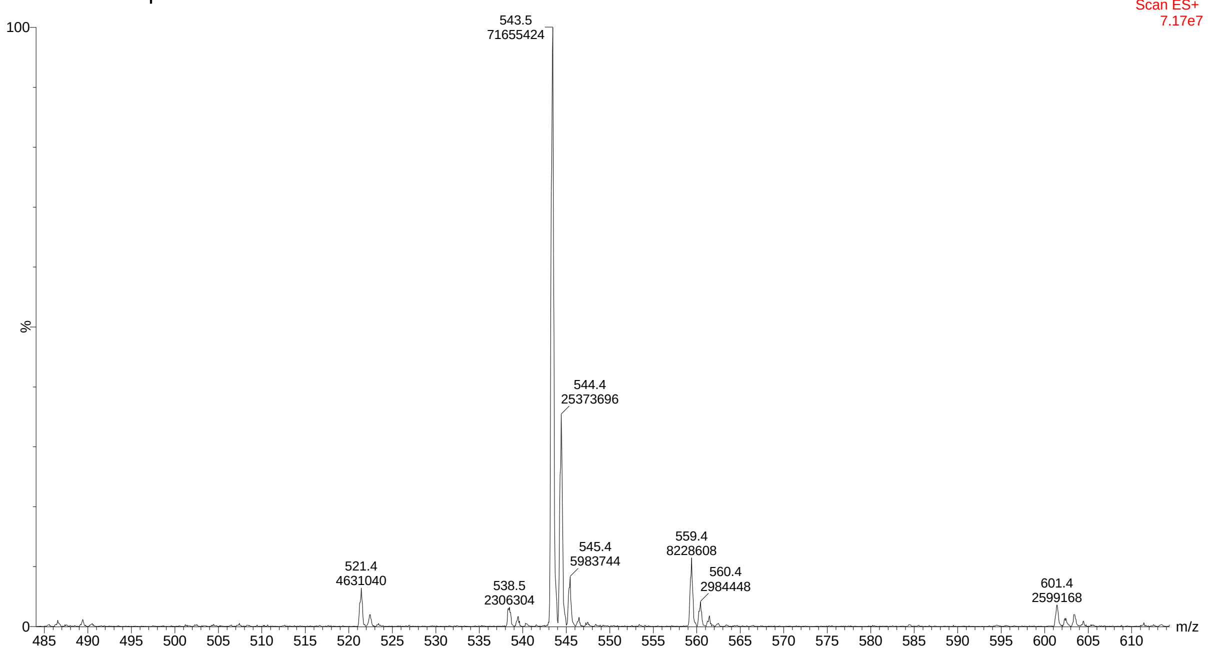

MS ESI$^+$ compound **4**

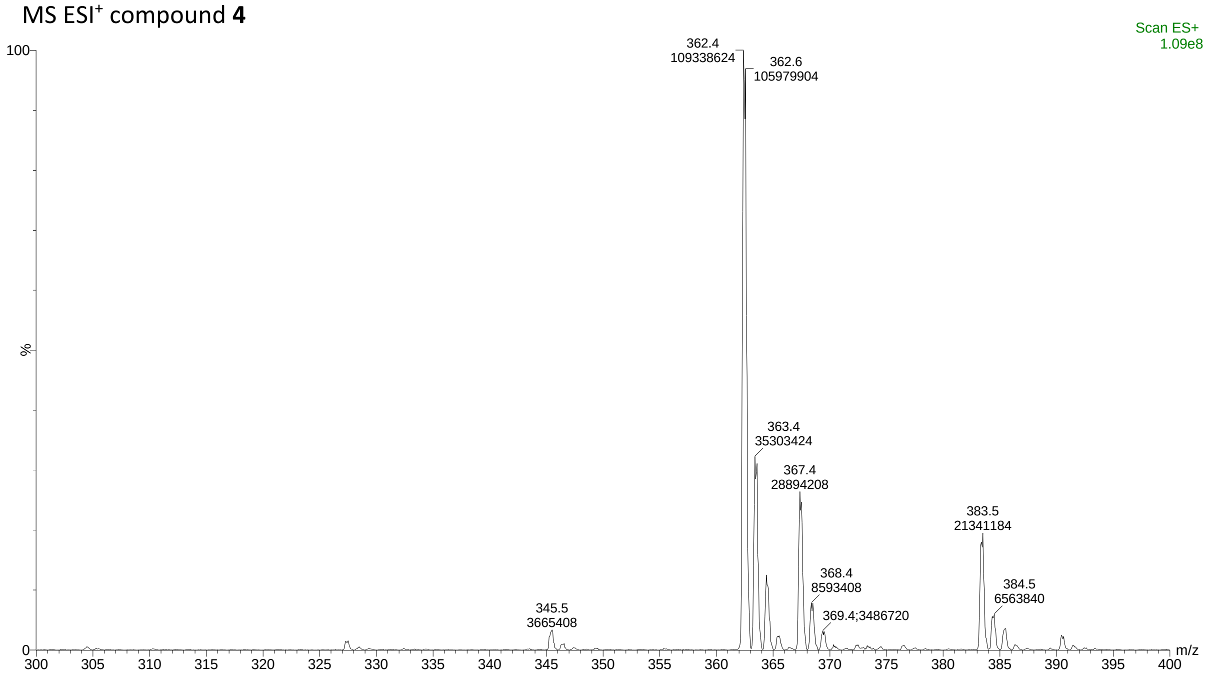

MS ESI$^+$ compound **6**

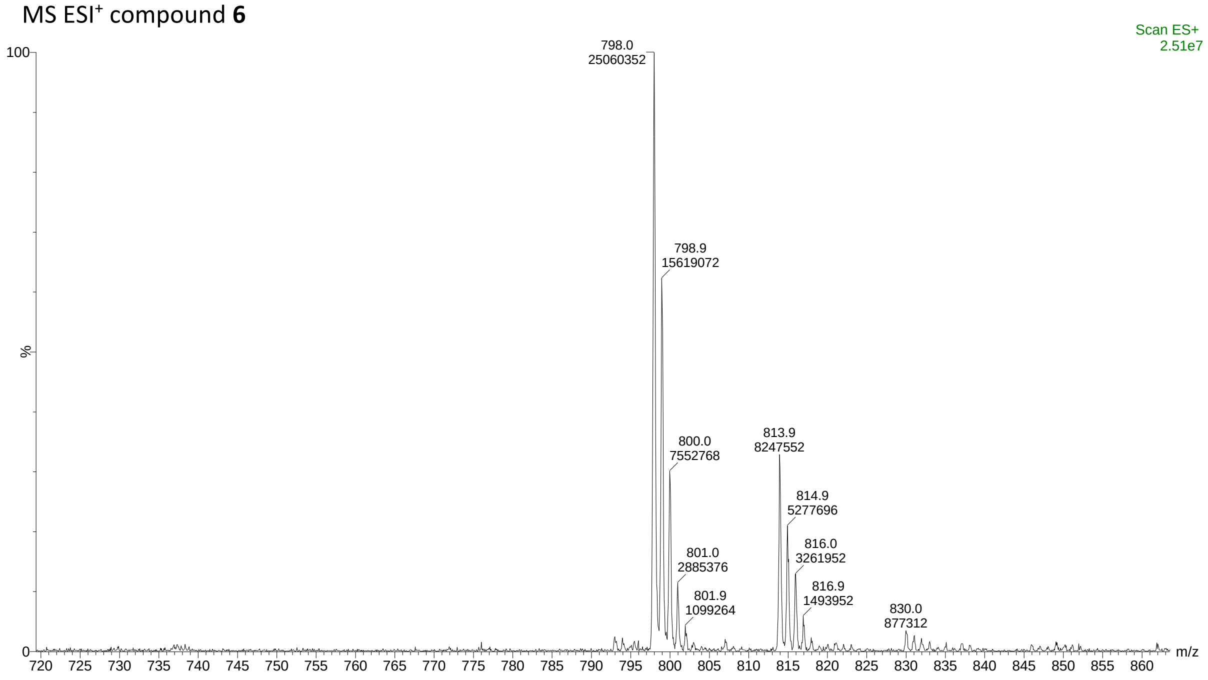

MS ESI$^+$ compound **8**

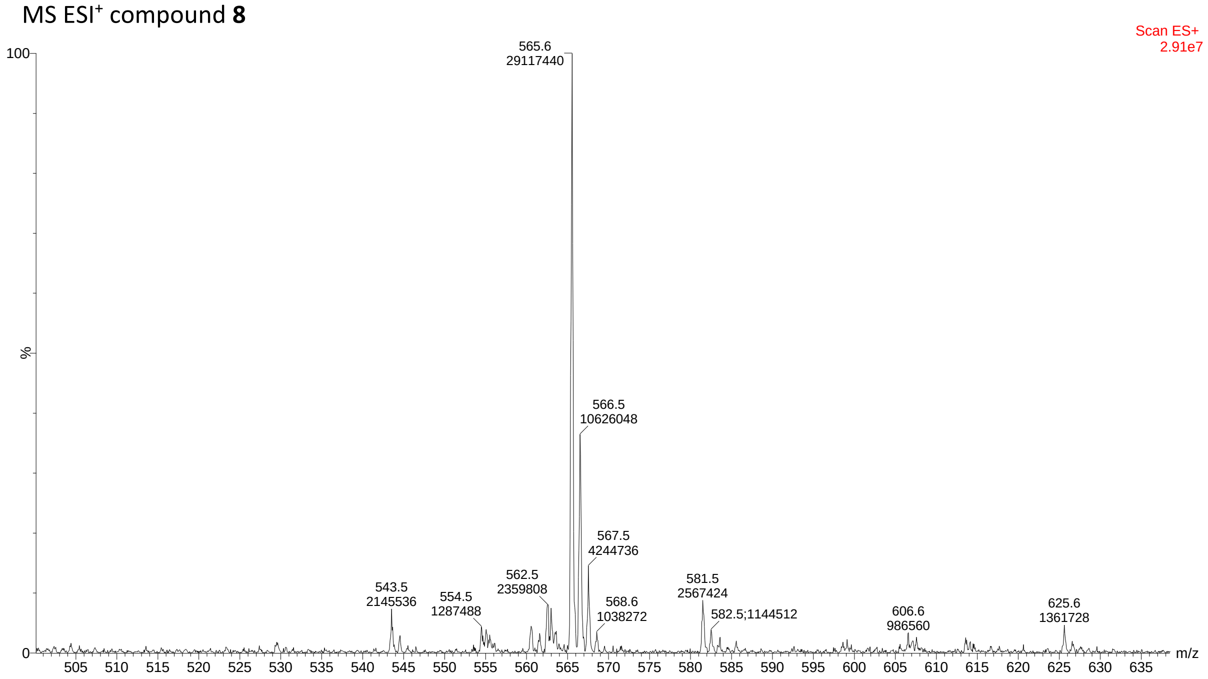

MS ESI^{+} compound **9**

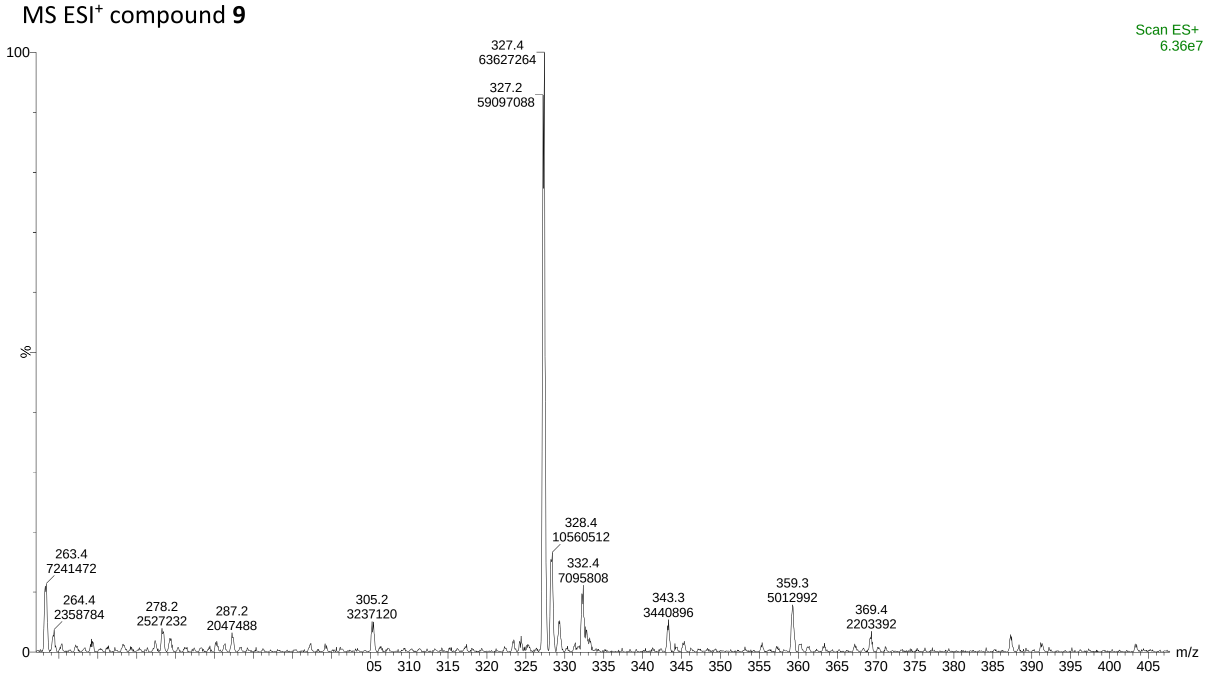

MS ESI^{-} compound **10**

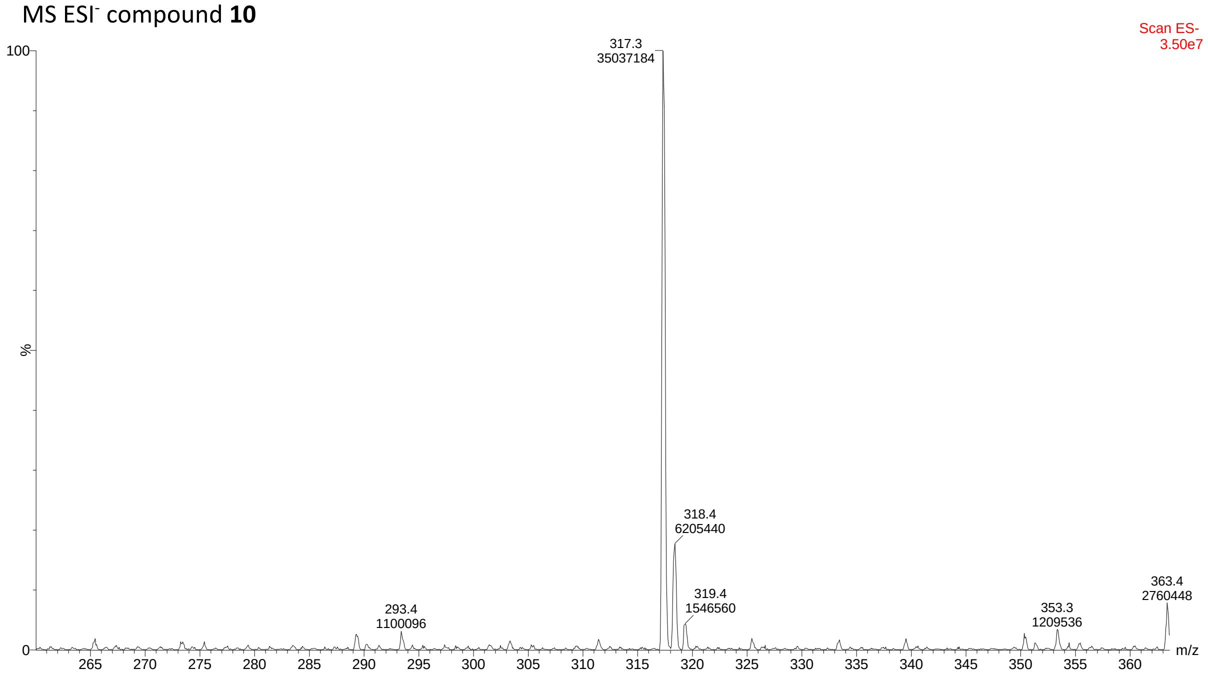

MS ESI$^-$ compound **12**

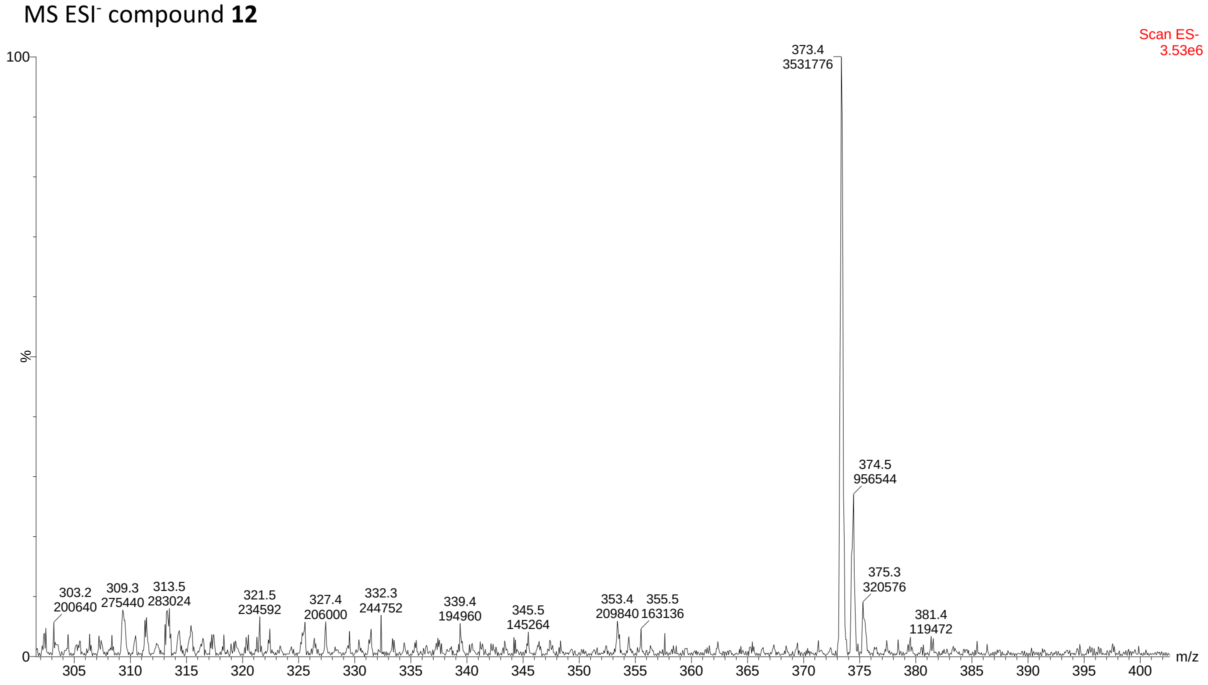

Supplement: Supplementary file 1 [file ijms-24-11258-s001.zip › ijms-2456821-supplementary.pdf]
